# Longitudinal Trends, Determinants, and Cardiometabolic Impact of Adherence to the Mediterranean Diet among Greek Adults

**DOI:** 10.3390/foods11162389

**Published:** 2022-08-09

**Authors:** Michael Georgoulis, Ekavi N. Georgousopoulou, Christina Chrysohoou, Christos Pitsavos, Demosthenes B. Panagiotakos

**Affiliations:** 1 Department of Nutrition and Dietetics, School of Health Sciences and Education, Harokopio University of Athens, 17676 Athens, Greece; 2 Medical School, Australian National University, Canberra 2601, Australia; 3 First Cardiology Clinic, Athens Medical School, National and Kapodistrian University of Athens, 11527 Athens, Greece

**Keywords:** Mediterranean diet, adherence, trends, trajectories, sustainability, cardiometabolic risk, socioeconomic status, lifestyle, nutrition transition, public health

## Abstract

Despite the well-established health benefits of the Mediterranean diet, there are signs that Mediterranean populations are deviating from this traditional pattern. We aimed to evaluate longitudinal changes in adherence to the Mediterranean diet, its determinants and health effects in a representative sample of the adult Greek population. This was a secondary analysis of the ATTICA epidemiological cohort study conducted in 2001/2002 and 2011/2012. The study sample consisted of 3042 men and women free of cardiovascular diseases living in Attica, Greece; of them, 2583 were followed-up for 10 years. Participants were evaluated in terms of sociodemographic, lifestyle and clinical parameters at baseline, and incidence of cardiometabolic diseases was recorded at follow-up. Dietary habits were assessed both at baseline and 10 years through a validated food frequency questionnaire and adherence to the Mediterranean diet was evaluated through the MedDietScore, based on which four trajectories were identified, i.e., low–low, low–high, high–low and high–high. During the study period, 45.6% of participants moved away from the Mediterranean diet (high–low), 9.0% moved closer (low–high), while 18.7% sustained a high adherence (high–high). Participants in the high–high trajectory were younger, mostly women, more physically active, had a higher socioeconomic status, and a more favorable body composition and cardiometabolic profile at baseline, and exhibited lower 10-year incidence rates of hyperlipidemia, hypertension, diabetes mellitus and cardiovascular disease compared to other trajectories (all *p*-values < 0.050). Adherence to the Mediterranean diet is declining among Greek adults. Staying close to the Mediterranean diet is associated with significant health benefits and should be a major target of public health strategies.

## 1. Introduction

Originally conceived by Ancel Keys in the landmark Seven Countries study [1,2], the term “Mediterranean diet” refers to the traditional dietary habits adopted in the olive-growing areas of the Mediterranean region during 1960s which were characterized by consumption of olive oil as the main source of dietary fat, a wide variety of plant-based foods (fruits, vegetables, bread, other forms of whole grains, legumes, nuts and seeds), moderate amounts of dairy products (cheese and yogurt), low-to-moderate amounts of fish and poultry, low amounts of red meat, and low-to-moderate quantities of wine accompanying meals [3,4]. Since then, the Mediterranean diet has been acknowledged as “Intangible Cultural Heritage of Humanity” by the United Nations Educational, Scientific and Cultural Organization (UNESCO) [5], while accumulated research over the past few decades has consistently shown that greater adherence to the Mediterranean diet is associated with profound health benefits. These include a reduction in total mortality, lower incidence of cardiovascular disease (CVD), diabetes mellitus, cancer and neurodegenerative diseases, as well as beneficial effects on body weight, lipidemic profile, glucose metabolism, blood pressure, inflammation and oxidative stress markers [6,7].

The health benefits of the Mediterranean diet can be attributed to its unique variety of foods and bioactive nutrients which act synergistically towards disease prevention. In specific, the plant-based foods of the Mediterranean diet, such as fruits, vegetables, whole grains, legumes, nuts and seeds, are rich in dietary fiber, micronutrients (vitamins and minerals) and several bioactive compounds (e.g., phenols, flavonoids, isoflavonoids, phytosterols, terpenes, phytic acid and phytoestrogens) which exert strong anti-oxidant effects [8]. Olive oil, a key feature of the Mediterranean-style diet, is abundant in monounsaturated fatty acids, polyphenolic compounds, squalene and α-tocopherol, and can explain a substantial part of the protective effects of the Mediterranean diet due to its ability to beneficially modify immune and inflammatory responses and contribute to reduced risk of CVD and cancer [9]. Moreover, polyunsaturated *n*-3 fatty acids found in fish have well-established hypotriglyceridemic, anti-arrhythmic, anti-inflammatory and anti-thrombotic properties, and play a vital role in the maintenance of neural functions and the prevention of certain neurocognitive disorders [10]. Other components of the Mediterranean diet that complete the puzzle of its health benefits include wine, which incorporates several micro-constituents (e.g., resveratrol, quercetin and gallic acid) that can improve lipid metabolism, glucose homeostasis and endothelial function [11], and fermented dairy products (cheese and yogurt) rich in probiotics (lactic acid bacteria) which can improve gastrointestinal health and the immune response of the host [12].

Despite the well-established health benefits of the Mediterranean diet, there is evidence of a gradual deviation from this dietary pattern within Mediterranean populations [13,14,15]. For instance, a longitudinal analysis of worldwide dietary data based on Food and Agriculture Organization Food Balance Sheets revealed that the mean Mediterranean Adequacy Index (computed by dividing the sum of the percentage of total energy from typical Mediterranean food groups by the sum of the percentage of total energy from non-typical Mediterranean food groups) decreased from 3.46 ± 1.28 in 1961–1965 to 2.03 ± 0.90 in 2000–2003 and 2.00 ± 0.93 in 2004–2011 within Mediterranean countries [13]. This shift in dietary habits is the result of the nutritional transition phenomenon, i.e., the gradual abandonment of traditional dietary habits and practices due to urbanization, increasing affluence and the progressive globalization of food supply [16]. Epidemiological data have shown low rates of and decreasing trends in the level of adherence to the Mediterranean diet in typical Mediterranean countries, including Greece [17,18,19], Spain [20,21,22], Portugal [23,24,25] and Italy [26,27,28], especially in younger individuals and population groups of low socioeconomic status. A decreasing adherence to the Mediterranean diet is parallel to an increasing adoption of a Western-type dietary pattern, characterized by high intakes of pre-packaged foods, refined grains, processed meat, sugar-sweetened beverages, sweets and other highly-processed energy-dense foods rich in salt, sugars and saturated fat; indicatively, mean global Western Diet Similarity Index (computed as the proportion of per-capita total calories from animal source foods plus oils and fats from plant sources and sweeteners) has increased from 40.57 ± 17.15 in 1993 to 42.24 ± 16.04 in 2013 [29]. This transition can explain, at least in part, the current public health epidemic of obesity and cardiometabolic morbidity within Mediterranean countries that were traditionally the birthplace of the Mediterranean diet [30,31].

Given the aforementioned, there is an urgent need to counter this tendency and implement evidence-based strategies to safeguard and re-promote the Mediterranean diet within Mediterranean populations. Using data from the ATTICA epidemiological cohort study (2001/2002–2011/2012), the aims of the present work were to evaluate longitudinal changes in the level of adherence to the Mediterranean diet in a representative sample of the adult Greek population, explore the cardiometabolic health impact of these changes, and identify the determinants of sustaining a high adherence to the Mediterranean diet. 

## 2. Materials and Methods

### 2.1. Study Design and Sample

This manuscript presents secondary analyses of the ATTICA cohort study, a prospective health- and nutrition-related survey, designed to evaluate a wide range of sociodemographic, lifestyle, clinical, biochemical and psychological parameters and explore their prognostic value on the incidence of CVD through periodic follow-up examinations in a representative sample of the adult Greek population. The study was conducted in accordance with the principles of the Declaration of Helsinki [32] and the study protocol was approved by the Ethics Committee of the First Cardiology Department of the National and Kapodistrian University of Athens (Approval Code: 017/01.05.2001, Approval Date: 1 May 2001). In brief, the baseline phase of the ATTICA study was carried out during 2001–2002 among adult men and women living in the province of Attica, Greece (78% urban municipalities), where Athens is the major metropolis, using a random multistage sampling procedure based on the age and sex distribution of the reference population, as well as the spatial distribution of the Attica region (27 cities sampled). Of the 4056 initially invited individuals, 117 were excluded based on a CVD diagnosis and 3042 CVD-free individuals (75% participation rate) agreed to participate and provided a signed written consent; of those, 1514 were males aged 46 ± 13 years (range: 18–87 years) and 1528 were females aged 45 ± 13 years (range: 18–89 years). At baseline, all participants were evaluated at their home or workplace through face-to-face interviews by a trained team of health professionals, including dietitians–nutritionists, cardiologists, general practitioners and nurses, using a standardized protocol (see details below). The study population was followed-up at 5 and 10 years after enrolment. For the purpose of the present secondary analyses, we combined baseline and 10-year follow-up dietary data to extract trajectories of adherence to the Mediterranean diet, test their association with sociodemographic, lifestyle, anthropometric, biochemical, psychological and clinical characteristics of the study population and explore their effects on the 10-year incidence of cardiometabolic outcomes, which were the main endpoints of the present work (see details below). Further information about the aims, design, sampling procedure and methodology of the ATTICA study can be found in previously published reports [33,34,35,36].

### 2.2. Baseline Evaluation

#### 2.2.1. Sociodemographic, Clinical and Biochemical Parameters

Participants were evaluated in terms of sociodemographic characteristics, i.e., age, sex, income, education, occupation and marital status through a detailed questionnaire as previously described [33,34,35,36]. Financial status was categorized based on the mean annual income over the last three years as: low—≤ EUR 8000; medium—EUR 8001–10,000; high—EUR 10,001–20,000; and very high—>EUR 20,000. Educational status was categorized as low, medium and high if total years of education (primary, secondary and tertiary) were 0–6, 7–13 and >14, respectively. Overall socioeconomic status (SES) was assessed through the integration of the years of education, mean annual income and occupation of each participant, and classified into low, middle and high [33,36]. Moreover, participants were physically examined by a physician, and a detailed medical history was obtained with information about the presence of cardiovascular risk factors, the use of relevant medications and family history of cardiometabolic diseases [33,35]. Arterial blood pressure was measured following a standardized protocol, and hypertension (HTN) was defined as blood pressure (SBP) ≥140 mmHg or diastolic blood pressure (DBP) ≥90 mmHg or reception of antihypertensive medication. Morning blood samples were also collected from participants at baseline after a 12 h fasting period, and serum total cholesterol (TC), high-density lipoprotein cholesterol (HDL-C), triglycerides (TG), glucose, insulin and high sensitivity C-reactive protein (hsCRP) were measured through appropriate assays as previously described [34,35]. Low-density lipoprotein cholesterol (LDL-C) was calculated using the Friedewald formula [37], while the homoeostasis model assessment of insulin resistance (HOMA-IR) was calculated according to Matthews et al. [38]. Hypercholesterolemia (HCL) was defined as TC > 200 mg/dL or the use of lipid-lowering agents, type 2 diabetes mellitus (T2DM) was diagnosed as fasting blood glucose ≥ 126 mg/dL or the use of antidiabetic agents and/or insulin [39], and the presence of the metabolic syndrome (MetS) was defined according to the NCEP ATP III definition [40].

#### 2.2.2. Anthropometric Indices and Lifestyle Habits

A detailed description of participants’ anthropometric and lifestyle evaluation can be found elsewhere [33,34,35,36]. In brief, body weight, height and waist circumference (WC) were measured following standard procedures and the body mass index (BMI) was calculated, based on which participants were classified as underweight, normal weight, overweight or obese [41]. Increased WC was defined as ≥102 cm for men and ≥88 cm for women according to international criteria [42]. In addition, the waist-to-height ratio (WHtR) was calculated and participants with WHtR > 0.5 were classified as centrally obese [43,44,45]. Dietary habits were assessed through the EPIC-Greek questionnaire [46], a 156-item semi-quantitative food-frequency questionnaire (FFQ) validated for the Greek population. Based on the FFQ data, habitual intake of individual foods and food groups was recorded, and adherence to the Mediterranean diet was evaluated through the Mediterranean Diet Score (MedDietScore) [47], ranging from 0 to 55, with higher values indicating a greater adherence to the Mediterranean diet. The short-form International Physical Activity Questionnaire (IPAQ) [48] was used to evaluate participants’ physical activity level. Based on the IPAQ data, weekly minutes of metabolic equivalent of tasks (METmin) were calculated and each participant was classified as minimally active, moderately active or highly active according to IPAQ scoring guidelines [49]. Regarding smoking habits, current smokers were defined as participants who smoked ≥1 cigarette/day, former smokers as those who had ceased smoking for ≥1 year prior to the evaluation, while never smokers as those with no history of tobacco use. Pack-years were also calculated for each participant as an index of total smoking exposure by multiplying smoking duration (in years) with the number of packs per day (assuming 20 cigarettes in a pack).

#### 2.2.3. Depression and Anxiety Symptoms

Depression levels were assessed using a translated and validated version of the Zung Self-Rating Depression Scale (ZDRS) [50]. The ZDRS consists of 20 items covering affective, psychological and somatic symptoms and the respondent is asked to specify the frequency with which the symptom is experienced on a 4-point Likert scale (from a little = 1 to most of the time = 4). The ZDRS score ranges from 20 to 80, with higher scores indicating more severe depression. Anxiety levels were assessed using the validated Greek translation of the State Anxiety sub-scale of the Spielberger State-Trait Anxiety Inventory (STAI) [51]. The STAI instrument includes a total of 40 questions (20 for trait anxiety and 20 for state anxiety), which are rated on a 4-point Likert scale (from almost never = 1 to almost always = 4). The 20-item State Anxiety sub-scale score ranges from 20 to 80, with higher values corresponding to a greater degree of anxiety symptoms.

### 2.3. Follow-Up Evaluation

The 10-year follow-up phase of the ATTICA study was carried out during 2011–2012 (mean follow-up: 8.41 years). Of the 3042 initially enrolled participants, 459 were lost to follow-up (could not be contacted or declined to undergo re-evaluation) and 2583 were re-evaluated (85% participation rate). At the follow-up examination, detailed information from participants’ medical records were retrieved (participants were asked to provide their medical records to the study investigators for review). Information was collected on vital status (death from any cause) and the development of cardiometabolic disorders, i.e., HCL, HTN, MetS, T2DM and fatal or non-fatal CVD. In addition, participants’ dietary habits were re-evaluated at 10 years using the same FFQ [46], and adherence to the Mediterranean diet was once again assessed through the MedDietScore [47].

### 2.4. Mediterranean Diet Trajectories

Both at baseline and the 10-year follow-up, participants were classified into two categories according to MedDietScore values, i.e., below the median = low adherence to the Mediterranean diet and equal or above the median = high adherence to the Mediterranean diet). Based on the change in the level of adherence to the Mediterranean diet (baseline vs. 10 years), four Mediterranean diet trajectories were identified, i.e., low–low (participants with low adherence at both evaluations), low–high (participants with low adherence at baseline and high adherence at 10 years), high–low (participants with high adherence at baseline and low adherence at 10 years) and high–high (participants with high adherence at both evaluations). In addition, differences in the MedDietScore values between baseline and the 10-year follow-up were calculated for each participant and the derived latent variable (ΔMedDietScore), was also used to test the research hypotheses. 

### 2.5. Statistical Analysis

Categorical variables are presented as absolute (relative) frequencies, while continuous variables are presented as mean ± standard deviation if normally distributed or as median (1st, 3rd quartile) if skewed. The Kolmogorov–Smirnov test was used to evaluate the normality of the distribution of numerical variables. Between-group differences were tested through the Pearson’s chi-squared test for categorical variables, the analysis of variance (ANOVA) for normally distributed numerical variables or the Kruskal–Wallis signed rank-test for skewed numerical variables. When differences between groups were significant, post-hoc comparisons were performed using the Bonferroni correction criterion to adjust for multiplicity. The incidence of HCL, HTN, MetS, T2DM and CVD was the main endpoint of the study. For HCL, HTN, MetS and T2DM, the crude incidence was calculated as the ratio of new cases to the total number of participants in the follow-up who were free of the corresponding condition at baseline, since only the diagnosis and not the exact time of onset of these outcomes was recorded. For CVD, incidence density was calculated using person-years as the denominator. Multiple logistic regression analysis was used to identify determinants of sustained high adherence to the Mediterranean diet, using the high–high trajectory as the dependent variable (compared to all other three trajectories, i.e., low–low, high–low and low–high); results are presented as odds ratios (OR) with their corresponding 95% confidence intervals (CI). Multiple linear regression analysis was also used to test potential determinants of ΔMedDietScore (10 years vs. baseline) as a continuous variable (b-coefficients and standard errors were extracted). Interactions between independent variables were checked and when significant, analyses were stratified. All reported *p*-values were based on two-sided tests and compared to a significance level of 5%. The STATA version 15 (StataCorp. 2017. College Station, TX, USA) was used for all analyses.

## 3. Results

The study’s sample consisted of 2583 participants (49.9% males) with a mean age of 46 ± 14 years. Mean MedDietScore values in the total study sample were 25.7 ± 6.7 at baseline and 25.0 ± 6.6 at the 10-year follow-up, indicative of a moderate level of adherence to the Mediterranean diet (MedDietScore range: 0–55). Based on the longitudinal change of MedDietScore values between baseline and 10 years, 687 participants (26.6%) were classified in the low–low Mediterranean diet trajectory (sustained low adherence over 10 years), 233 (9.0%) in the low–high trajectory (increasing adherence over 10 years), 1179 (45.6%) in the high–low trajectory (decreasing adherence over 10 years) and 484 (18.7%) in the high–high trajectory (sustained high adherence over 10 years).

Participants’ baseline sociodemographic characteristics according to trajectories of adherence to the Mediterranean diet are shown in Table 1. A significant difference between trajectories was observed in age, with participants in the high–low and high–high trajectories being younger compared to those in the low–low and low–high trajectories. Participants in the high–low and high–high trajectories were mostly female, while male gender was dominant in the low–low and low–high trajectories. Educational level and marital status also differed significantly among trajectories, with the high–low and high–high trajectories being characterized by higher total years of education and a higher prevalence of single (non-married) individuals compared to the low–low and low–high trajectories. A higher financial status was also observed in the low–high trajectory compared to others, while the percentage of participants with low total SES was significantly lower in the high–high trajectory compared to all other trajectories.

Significant differences between Mediterranean diet trajectories were also observed in baseline anthropometric parameters and lifestyle habits (Table 2). Specifically, participants in the high–low and high–high trajectories exhibited lower mean BMI, WC and WHtR values, and lower rates of obesity (BMI ≥ 30 kg/m^2^), increased WC (≥102 cm for males and ≥88 cm for females) and central obesity (WHtR > 0.5), compared to the low–low and low–high trajectories. For all the above-mentioned anthropometric parameters, the high–high trajectory presented the most beneficial status, with significant differences compared to all other trajectories. The high–high trajectory was also characterized by a higher physical activity level, expressed as MET-min/week, compared to the low–low trajectory. The percentage of current smokers was higher in the high–low and high–high trajectories compared to the low–low one, however the distribution of ever smokers and median pack-years were similar among trajectories. Regarding symptoms of depression and anxiety (Table 2), the low–high trajectory exhibited the lowest mean values of ZDRS and STAI; however. between-group differences were significant only for mean ZDRS when comparing the low–high trajectory to the high–low and high–high trajectories.

Participants’ baseline cardiometabolic profile according to trajectories of adherence to the Mediterranean diet is shown in Table 3. A consistent pattern was observed for all cardiometabolic indices, with participants in the low–low and low–high trajectories presenting similar values, those in the high–low and high–high trajectories exhibiting a more favorable profile compared to those in the low–low and low–high trajectories, and those in the high–high trajectory presenting an even more beneficial profile compared to those in the high–low trajectory. Regarding medical status (Table 3), baseline prevalence of HCL, HTN, MetS and T2DM was lower in the high–low and high–high trajectories compared to the low–low and low–high ones, while individuals in the high–high trajectory exhibited lower rates of cardiometabolic diseases compared to the high–low trajectory. A longitudinal increase in the prevalence of cardiometabolic morbidity was observed within all four Mediterranean diet trajectories over the 10-year follow-up period, and a similar pattern to the baseline for between-group differences was observed at 10 years.

During the 10-year follow-up period, 352 new HCL cases, 320 new HTN cases, 914 new MetS cases and 191 new T2DM cases were recorded, yielding a crude incidence of 33.7%, 27.7%, 44.6% and 12.9%, respectively. Regarding CVD, 317 new cases were recorded (198 in men and 119 in women), which corresponded to a crude incidence of 15.7% (19.7% in men and 11.7% in women) and an annual incidence of 146 new cases per 10,000 participants (182 per 10,000 men and 110 per 10,000 women). Crude incidence rates of cardiometabolic diseases per Mediterranean diet trajectory are presented in Table 3. Pairwise comparisons between trajectories revealed that the low–low and low–high trajectories had similar incidence rates for all cardiometabolic diseases, while the high–low trajectory exhibited a lower incidence of MetS, T2DM and CVD compared to the low–low and low–high trajectories, and the high–high trajectory exhibited the lowest incidence of all cardiometabolic outcomes compared to other trajectories. CVD incidence, expressed as new cases per 10,000 person-years, was 266 in the low–low trajectory, 275 in the low–high trajectory, 93 in the high–low trajectory and 38 in the high–high trajectory.

Various baseline parameters were finally explored as potential determinants of the long-term sustainability of high adherence to the Mediterranean diet in multiple logistic regression analysis (Table 4). In the total sample, age (OR: 0.942, 95% CI: 0.927–0.956 for every 1-year increase), sex (OR: 0.163, 95% CI: 0.110–0.241 for males) and BMI (OR: 0.756, 95% CI: 0.715–0.799 for every 1 kg/m^2^ increase) emerged as important determinants of being in the high–high Mediterranean diet trajectory (versus all other three trajectories), whereas SES, smoking habits and physical activity level were not significant predictors. When analyses were stratified according to age, the most important determinants of sustaining a high adherence to the Mediterranean diet in participants aged <40 years were female sex (81% higher odds), low BMI (22% lower odds for every 1 kg/m^2^ increase) and high physical activity level (1.4% higher odds for every 100-MET-min/week increase). Accordingly, in participants aged ≥40 years, low BMI (48% lower odds for every 1 kg/m^2^ increase) and medium/high SES (approximately 5 times greater odds compared to low SES) emerged as the strongest predictors of being in the high–high Mediterranean diet trajectory (Table 4). The aforementioned results were also confirmed in linear regression analysis models with ΔMedDietScore as the dependent variable (data not shown).

## 4. Discussion

Our results indicate that over a 10-year follow-up, approximately half of the adult Greek population experienced a deterioration of dietary habits and moved away from the Mediterranean diet, less than 10% improved their dietary habits and approximately 20% sustained a high adherence to the Mediterranean diet. Individuals who sustained a high level of adherence to the Mediterranean diet were younger, mostly women, had a high SES, as well as a favorable body composition and cardiometabolic profile at baseline, and exhibited significantly lower 10-year incidence rates of cardiometabolic diseases. Among various parameters, the most important determinants of sustaining a high adherence to the Mediterranean diet were female sex, low BMI and high physical activity level in individuals aged <40 years, and low BMI and high SES in those aged ≥40 years.

In line with our findings, epidemiological studies both in adult and paediatric populations have pointed out that the diets of Mediterranean populations are gradually moving away from traditional patterns [13,14,15]. Several factors have been proposed as causative of this transition. For example, the enhanced commercial availability of food, the urbanization of life and the overall improvement in socioeconomic conditions in Europe have increased food and energy supply and made food (especially that of animal origin) more affordable to most households [22,52,53]. Despite the overall socioeconomic improvement compared to the original observations of the Mediterranean diet in 1960s, the more recent economic crisis (2008–2009) in Europe is also thought to have contributed to decreasing rates of adherence to the Mediterranean diet, due to an increase in the prices of some food items typical of the Mediterranean diet pyramid, such as fruits, vegetables, whole grains and fish, compared to the more affordable prices of refined grains, sweets and snacks [54]. Moreover, cultural interactions across populations in Mediterranean regions secondary to the increase in migration and tourism observed during the last decades has also probably led to a partial replacement of traditional Mediterranean foods and habits with Western-type ones [55]. Last but not least, the lower amount of available time for shopping and cooking due to the more demanding and stressful modern lifestyle is also thought to have contributed to the gradual abandonment of traditional Mediterranean dietary practices (food recipes, food processing and cooking techniques, convivial-social meals, etc.) and to a shift to eating alone, less time-consuming meals and fast food [56]. 

An interesting finding of our study is that individuals who initially adhered closely to the Mediterranean diet but gradually moved away from it exhibited a lower incidence of cardiometabolic diseases compared to those who sustained a low adherence over time. This observation suggests that an initial high adherence to the Mediterranean diet is associated with a more favorable health status even if dietary habits deteriorate over time, compared to a long-term low adherence. However, it is also possible that a 10-year follow-up was not sufficient to detect the longer-term cardiometabolic burden associated with worsening dietary habits. Contrariwise, the same subgroup of participants exhibited a significantly higher incidence of cardiometabolic diseases comparted to those who sustained a high adherence to the Mediterranean diet over 10 years of follow-up. This observation supports that moving away from a prudent diet has a detrimental health effect and highlights the need for public health interventions in Mediterranean populations to safeguard traditional healthy dietary habits and combat the epidemic of cardiometabolic diseases. Our results are in line with those of previous studies both in Mediterranean and non-Mediterranean populations suggesting that prospective decreases in the level of adherence to the Mediterranean diet are associated with detrimental changes in cardiometabolic risk factors [57,58] and further confirm the well-established and universally recognized health benefits of the Mediterranean diet [6,7].

A minority (9%) of the study population increased their level of adherence to the Mediterranean diet over time. This low percentage probably reflects the difficulty in improving and sustaining beneficial dietary changes in the adult population, which has been highlighted in numerous previous studies [59,60,61,62]. Participants in the low–high Mediterranean diet trajectory exhibited a higher baseline financial status compared to participants in the low–low Mediterranean diet trajectory. This can explain, at least in part, the longitudinal improvement in their dietary habits towards the Mediterranean diet, in line with previous evidence of a positive correlation between SES and healthy dietary choices [63,64,65]. Moreover, the low–high trajectory exhibited lower ZDRS values compared to the high–low trajectory, indicating that a good psychological status can beneficially impact longitudinal changes in dietary habits and vice versa [66,67,68]. Despite the improvement in dietary habits in the low–high trajectory, this was not translated into a lower incidence of cardiometabolic diseases compared to the low–low trajectory. This could be attributed to the higher age distribution of the low–high trajectory group and to its detrimental biochemical profile and medical status at baseline. In other words, it is possible that participants classified in the low–high trajectory represented a group of older individuals with a longer life-time duration of poor dietary habits, the health impact of which would be difficult to counteract later in life, or individuals who improved their dietary habits to ameliorate an impaired health status (e.g., those who self-improved dietary habits or received dietary counselling due to a high cardiometabolic burden).

Maintaining a lifelong adherence to a prudent diet is considered the cornerstone of cardiometabolic disease prevention and health promotion along with other healthy lifestyle behaviors [69]. Several dietary patterns have emerged as health promoting, however the ideal diet for a population is also relevant to its chances of sustainability based on food availability, natural resources, cost and the sociocultural characteristics of each region. Although the Mediterranean diet was originally identified as the collection of traditional dietary habits of the populations of the countries surrounding the Mediterranean Sea, our findings suggest that only a minority (approximately 19%) of the adult population in Greece, a typical Mediterranean country, have sustained a high adherence to the Mediterranean diet during 2000s and early 2010s. As expected, this subgroup of the population exhibited the most beneficial health status both at baseline and 10 years and a much lower incidence of cardiometabolic diseases compared to individuals who improved or worsened their dietary habits, and especially those who remained away from the Mediterranean diet over the same time period. Our results are in line with previous data showing that a high baseline level of adherence to the Mediterranean diet significantly decreases 10-year CVD risk, after taking into account various sociodemographic, lifestyle, and clinical factors [70]. The mechanisms explaining the cardiometabolic benefits of the Mediterranean diet have been extensively studied and include beneficial effects on blood lipids, enhancement of insulin sensitivity, amelioration of chronic low-grade inflammation and oxidative stress, improvement of endothelial function and antithrombotic properties; these effects are attributable to the synergistic action of bioactive ingredients present in typical Mediterranean foods, such as dietary fiber, antioxidants (e.g., polyphenols and resveratrol) and unsaturated fatty acids [71].

Among various parameters, the most important determinants of sustaining a high level of adherence to the Mediterranean diet were female sex, low BMI and high physical activity level in individuals aged <40 years at baseline, and low BMI and high SES in those aged ≥40 years at baseline. These data are in line with previous publications of our research team showing that adherence to the Mediterranean diet is greater among females compared to males, physically active individuals compared to sedentary, high-SES individuals compared to low-SES, and normal-weight individuals compared to overweight or obese [72,73], and seem to persist longitudinally but also to differentiate according to age. For instance, it is possible that physical activity correlates with a healthy diet among young individuals but not among older ones, in whom sedentariness is highly prevalent due to age-related physical limitations, while SES might have a greater impact on dietary choices among older individuals who experience age-related detrimental changes in functionality, productivity, occupational and financial status. When interpreted in a different way, our findings highlight two distinct population groups with a need for efficient public health interventions to increase adherence to the Mediterranean diet: younger overweight/obese sedentary men and older individuals of low SES.

Previously published data from the ATTICA study have provided information on the dietary habits of the Greek population showing a moderate adherence to the Mediterranean diet during 2001–2002 (baseline phase of the study) [72], and have revealed the protective role of baseline high adherence to the Mediterranean diet on 10-year CVD incidence [70]. In this secondary analysis of the ATTICA study, we combined baseline and 10-year dietary data to extract Mediterranean diet trajectories and explored their relationship with population characteristics and 10-year incidence of cardiometabolic diseases. The present study is among the few that have so far explored longitudinal trends of adherence to the Mediterranean diet in relation to sociodemographic, lifestyle, psychological, biochemical and clinical parameters over 10 years of follow-up. The prospective design of the study, the evaluation of longitudinal trends of adherence to the Mediterranean diet, the inclusion of an adequate sample of the Greek adult population, as well as the comprehensive assessment of participants’ baseline characteristics that allowed a detailed comparison of the phenotypic differences of different Mediterranean diet trajectories, are strong points of the present work. The main limitation of our study is that only medical status and dietary habits were assessed both at baseline and the 10-year follow-up, therefore we could not evaluate the impact of longitudinal changes in other characteristics of the study population (e.g., weight status, physical activity or SES) on long-term adherence to the Mediterranean diet. Additional limitations include the assessment of SES indices, lifestyle habits and psychological symptoms through questionnaires which can be susceptible to errors, such as recall bias, although all tools used were validated and implemented by trained health professionals according to a standardized protocol to ensure maximum accuracy; and the study sample, which was large and randomly selected from the Attica region (hosting about the 50% of the Greek population) but does not permit the generalization of our findings to the whole Greek population, since several regions of the country were missing from the sampling, or to other Mediterranean populations. Last but not least, it must be noted that the four groups identified based on Mediterranean diet trajectories were unequal in terms of number of subjects (*n* = 687 in the low–low, *n* = 233 in the low–high, *n* = 1179 in the high–low and *n* = 484 in the high–high trajectory). However, the size of these groups reflects the actual trends of adherence to the Mediterranean diet in our study sample over a 10-year follow-up period, and highlights a gradual abandonment of the Mediterranean diet within the adult Greek population, similarly to the data reported in other Mediterranean countries.

## 5. Conclusions

In conclusion, adherence to the Mediterranean diet is gradually declining among the adult population of Greece, in line with the nutrition transition phenomenon evident in Mediterranean populations, and is associated with a detrimental health impact. Staying close to the Mediterranean diet in the long-term is associated with significant cardiometabolic health benefits and should constitute a major target of public health strategies. Specific groups of the population, i.e., younger overweight/obese sedentary men and older individuals of low SES, might be more likely to deviate from the Mediterranean diet and could benefit more from healthy dietary interventions. The present findings should be confirmed in other Mediterranean populations of different background and could serve as the basis for public health policies to further disseminate the health benefits of and promote lifelong adherence to the Mediterranean diet.

## Figures and Tables

**Table 1:** Baseline sociodemographic characteristics of the study population according to trajectories of adherence to the Mediterranean diet (*n* = 2583).

	Mediterranean Diet Trajectories				*p*-Value *
	Low–Low (*n* = 687)	Low–High (*n* = 233)	High–Low (*n* = 1179)	High–High (*n* = 484)	
Age, years	55.5 ± 13.5 ^a^	54.7 ± 10.7 ^a^	43.2 ± 12.0 ^b^	35.2 ± 10.0 ^c^	<0.001
Age group, *n* (%)					
<35 years 35–65 years >65 years	49 (7.1) ^a^ 442 (64.3) ^a^ 196 (28.5) ^a^	4 (1.7) ^b^ 187 (80.3) ^b^ 42 (18.0) ^b^	284 (24.1) ^c^ 842 (71.4) ^c^ 53 (4.5) ^c^	240 (49.6) ^d^ 243 (50.2) ^d^ 1 (0.2) ^d^	<0.001
Males, *n* (%)	500 (72.8) ^a^	190 (81.5) ^b^	524 (44.4) ^c^	76 (15.7) ^d^	<0.001
Marital status, *n* (%)					
never married married divorced widowed	64 (9.3) ^a^ 560 (81.6) ^a^ 25 (3.6) 38 (5.5) ^a^	13 (5.6) ^a^ 208 (89.3) ^a^ 4 (1.7) 8 (3.4) ^a,b^	257 (21.8) ^b^ 839 (71.2) ^b^ 47 (4.9) 36 (3.1) ^b^	206 (42.6) ^c^ 260 (53.7) ^c^ 14 (2.9) 4 (0.8) ^c^	<0.001
Education, years	11.0 ± 4.0 ^a^	11.6 ± 3.9 ^a^	12.3 ± 3.6 ^b^	13.3 ± 3.0 ^c^	<0.001
Educational level, *n* (%)					
low medium high	202 (29.4) ^a^ 301 (43.8) 184 (26.8) ^a^	58 (24.9) ^a^ 103 (44.2) 72 (30.9) ^a,b^	190 (16.1) ^b^ 572 (48.5) 417 (35.4) ^b^	32 (6.6) ^c^ 229 (47.4) 223 (46.0) ^c^	<0.001
Financial status, *n* (%)					
low medium high very high	113 (16.4) ^a^ 226 (32.9) 281 (40.9) ^a^ 67 (9.8) ^a^	17 (7.6) ^b^ 67 (28.7) 104 (44.6) ^a^ 45 (19.1) ^b^	258 (21.9) ^a,c^ 380 (32.2) 403 (34.2) ^a,b^ 138 (11.7) ^a,b^	136 (28.1) ^c^ 184 (38.0) 125 (25.8) ^b^ 39 (8.1) ^a^	<0.001
SES, *n* (%)					
low medium high	165 (24.0) ^a^ 345 (50.2) 177 (25.8) ^a^	44 (19.1) ^a,b^ 113 (48.4) 76 (32.5) ^a,b^	157 (13.3) ^b^ 641 (54.4) 381 (32.3) ^a,b^	28 (5.8) ^c^ 266 (54.9) 190 (39.3) ^b^	<0.001

Data are presented as absolute (relative) frequency for categorical variables and mean ± standard deviation for normally distributed numerical variables. * *p*-values for comparisons between Mediterranean diet trajectories, as derived from the Pearson’s chi-squared test for categorical variables, the analysis of variance (ANOVA) for normally distributed numerical variables or the Kruskal–Wallis signed rank-test for skewed numerical variables. ^a–d^ Different superscripts indicate significant between-group differences (*p*-value < 0.050) according to post hoc pairwise comparisons adjusted by the Bonferroni correction for multiple tests. SES, socioeconomic status.

**Table 2:** Baseline anthropometric indices, lifestyle habits, and symptoms of depression and anxiety of the study population according to trajectories of adherence to the Mediterranean diet (*n* = 2583).

	Mediterranean Diet Trajectories				*p*-Value *
	Low–Low (*n* = 687)	Low–High (*n* = 233)	High–Low (*n* = 1179)	High–High (*n* = 484)	
**Anthropometric indices**					
BMI, kg/m^2^	29.5 ± 4.2 ^a^	29.9 ± 4.2 ^a^	25.6 ± 3.3 ^b^	22.4 ± 2.5 ^c^	<0.001
BMI status, *n* (%)					
underweight normal-weight overweight obese	1 (0.1) ^a^ 69 (10.0) ^a^ 354 (51.5) ^a^ 263 (38.4) ^a^	0 (0.0) ^a^ 22 (9.4) ^a^ 108 (46.4) ^a^ 103 (44.2) ^a^	13 (1.1) ^a^ 488 (41.4) ^b^ 581 (49.3) ^a^ 97 (8.2) ^b^	21 (4.3) ^b^ 396 (81.8) ^c^ 64 (13.3) ^b^ 3 (0.6) ^c^	<0.001
WC, cm					
males females	102.8 ± 11.7 ^a^ 97.0 ± 14.5 ^a^	103.8 ± 11.2 ^a^ 99.4 ± 12.4 ^a^	93.3 ± 11.2 ^b^ 83.2 ± 11.8 ^b^	83.9 ± 9.2 ^c^ 76.3 ± 9.8 ^c^	<0.001 <0.001
Increased WC, *n* (%)	460 (67.0) ^a^	169 (72.5) ^a^	545 (46.3) ^b^	109 (22.6) ^c^	<0.001
WHtR	0.60 ± 0.07 ^a^	0.60 ± 0.07 ^a^	0.52 ± 0.07 ^b^	0.46 ± 0.06 ^c^	<0.001
Central obesity, *n* (%)	513 (74.7) ^a^	191 (82.0) ^a^	656 (62.1) ^b^	94 (19.4) ^c^	<0.001
**Lifestyle habits**					
Current smokers, *n* (%)	250 (36.4) ^a^	91 (39.1) ^a,b^	520 (44.1) ^b^	223 (46.2) ^b^	0.002
Ever smokers, *n* (%)	373 (54.3)	145 (62.2)	644 (54.7)	259 (53.6)	0.140
Pack-years	540 (223, 900) ^a^	600 (300, 1020) ^a^	320 (143, 600) ^b^	180 (60, 375) ^c^	<0.001
Energy intake, kcal/d	2499 ± 975 ^a^	2385 ± 883 ^a,b^	2424 ± 951 ^a^	2109 ± 879 ^b^	0.001
MET-min/week	231 (149, 1519) ^a^	231 (149, 1377) ^a^	198 (149, 735) ^a^	330 (149, 1406) ^b^	0.050
Physical activity status, *n* (%)					
minimally active moderately active highly active	415 (60.4) 107 (15.6) ^a^ 165 (24.0)	137 (58.8) 48 (20.6) ^a,b^ 48 (20.6)	726 (61.6) 210 (17.8) ^a,b^ 243 (20.6)	278 (57.5) 113 (23.3) ^b^ 93 (19.2)	0.022
**Depression and anxiety symptoms**					
ZDRS (20–80)	34.5 ± 7.2 ^a,b^	33.1 ± 5.5 ^a^	35.6 ± 7.8 ^b^	36.2 ± 7.5 ^b^	0.028
STAI (20–80)	40.6 ± 11.6	38.8 ± 11.4	41.2 ± 11.4	40.6 ± 12.5	0.524

Data are presented as absolute (relative) frequency for categorical variables, mean ± standard deviation for normally distributed numerical variables and median (1st, 3rd quartile) for skewed numerical variables. * *p*-values for comparisons between Mediterranean diet trajectories, as derived from the Pearson’s chi-squared test for categorical variables, the analysis of variance (ANOVA) for normally distributed numerical variables or the Kruskal–Wallis signed rank-test for skewed numerical variables. ^a–c^ Different superscripts indicate significant between-group differences (*p*-value < 0.050) according to post hoc pairwise comparisons adjusted by the Bonferroni correction for multiple tests. BMI, body mass index; STAI, State-Trait Anxiety Inventory; WC, waist circumference; WHtR, waist-to-height ratio; ZDRS, Zung Self-Rating Depression Scale.

**Table 3:** Baseline biochemical indices, blood pressure and medical status, as well as 10-year medical status and incidence of cardiometabolic diseases of the study population according to trajectories of adherence to the Mediterranean diet (*n* = 2583).

	Mediterranean Diet Trajectories				*p*-Value *
	Low–Low (*n* = 687)	Low–High (*n* = 233)	High–Low (*n* = 1179)	High–High (*n* = 484)	
**Cardiometabolic indices**					
Glucose, mg/dL	93 (85, 106) ^a^	97 (87, 108) ^a^	89 (80, 97) ^b^	86 (78, 94) ^c^	<0.001
Insulin, μU/ml	13.6 (12.6, 14.9) ^a^	14.1 (12.9, 15.3) ^a^	12.4 (11.5, 13.6) ^b^	11.5 (10.8, 12.3) ^c^	<0.001
HOMA-IR	3.12 (2.6, 3.9) ^a^	3.33 (2.77, 4.09) ^a^	2.72 (2.31, 3.20) ^b^	2.44 (2.10, 2.87) ^c^	<0.001
TC, mg/dL	201 (176, 230) ^a^	198 (177, 225) ^a^	190 (163, 219) ^b^	174 (153, 202) ^c^	<0.001
LDL-C, mg/dL	129 (107, 153) ^a^	126 (110, 150) ^a^	119 (97, 146) ^b^	106 (85, 131) ^c^	<0.001
HDL-C, mg/dL	43 (37, 51) ^a^	42 (35, 48) ^a^	48 (40, 57) ^b^	52 (44, 61) ^c^	<0.001
TG, mg/dL	123 (91, 172) ^a^	124 (93, 182) ^a^	96 (67, 140) ^b^	73 (54, 100) ^c^	<0.001
hs-CRP, mg/L	1.43 (0.66, 3.08) ^a^	1.51 (0.67, 2.95) ^a^	1.05 (0.47, 2.25) ^b^	0.60 (0.28, 1.33) ^c^	<0.001
SBP, mm Hg	132 ± 19 ^a^	131 ± 17 ^a^	122 ± 17 ^b^	112 ± 14 ^c^	<0.001
DBP, mm Hg	83 ± 11 ^a^	84 ± 11 ^a^	79 ± 11 ^b^	72 ± 10 ^c^	<0.001
**Medical status**					
Family history of HCL, *n* (%)	208 (66.9) ^a,b^	77 (58.3) ^a^	479 (72.6) ^b^	225 (76.5) ^b^	<0.001
HCL, *n* (%)-baseline	342 (49.8) ^a^	114 (48.9) ^a,b^	469 (39.8) ^b^	125 (25.9) ^c^	<0.001
HCL, *n* (%)-10 years	410 (59.7) ^a^	134 (57.5) ^a,b^	553 (46.9) ^b^	155 (32.0) ^c^	<0.001
Family history of HTN, *n* (%)	86 (12.5)	36 (15.5)	181 (15.4)	82 (16.9)	0.174
HTN, *n* (%)-baseline	314 (45.7) ^a^	103 (46.8) ^a^	297 (27.1) ^b^	41 (8.5) ^c^	<0.001
HTN, *n* (%)-10 years	397 (57.8) ^a^	128 (54.9) ^a^	392 (33.2) ^b^	62 (12.8) ^c^	<0.001
MetS, *n* (%)-baseline	238 (34.6) ^a^	97 (41.6) ^a^	181 (15.4) ^b^	14 (2.9) ^c^	<0.001
MetS, *n* (%)-10 years	486 (70.7) ^a^	172 (73.8) ^a^	622 (52.8) ^b^	163 (33.7) ^c^	<0.001
Family history of T2DM, *n* (%)	169 (24.6)	78 (33.5)	304 (25.8)	128 (26.4)	0.775
T2DM, *n* (%)-baseline	104 (15.1) ^a^	34 (14.6) ^a^	56 (4.7) ^b^	3 (0.6) ^c^	<0.001
T2DM, *n* (%)-10 years	173 (25.2) ^a^	71 (30.5) ^a^	128 (10.9) ^b^	16 (3.3) ^c^	<0.001
Family history of CVD, *n* (%)	156 (22.7)	60 (25.8)	317 (26.9)	127 (26.2)	0.534
**10-year incidence of cardiometabolic diseases**					
HCL 10-year incidence, *n* (%)	114 (45.4) ^a^	37 (40.2) ^a,b^	155 (32.8) ^b^	46 (20.2) ^c^	<0.001
HTN 10-year incidence, *n* (%)	121 (48.0) ^a^	31 (33.7) ^a,b^	139 (25.4) ^b^	29 (11.0) ^c^	<0.001
MetS 10-year incidence, *n* (%)	248 (55.2) ^a^	75 (55.1) ^a^	442 (44.4) ^b^	149 (31.7) ^c^	<0.001
T2DM 10-year incidence, *n* (%)	69 (19.2) ^a^	37 (25.5) ^a^	72 (10.4) ^b^	13 (4.6) ^c^	<0.001
CVD 10-year incidence, *n* (%)	153 (30.1) ^a^	56 (32.6) ^a^	93 (10.0) ^b^	15 (3.7) ^c^	<0.001

Data are presented as absolute (relative) frequency for categorical variables, mean ± standard deviation for normally distributed numerical variables and median (1st, 3rd quartile) for skewed numerical variables. * *p*-values for the comparisons between Mediterranean diet trajectories, as derived from the Pearson’s chi-squared test for categorical variables, the analysis of variance (ANOVA) for normally distributed numerical variables or the Kruskal–Wallis signed rank-test for skewed numerical variables. ^a–c^ Different superscripts indicate significant between-group differences (*p*-value < 0.050) according to post hoc pairwise comparisons adjusted by the Bonferroni correction for multiple tests. HCL, hypercholesterolemia; HDL-C, high-density lipoprotein cholesterol; CVD, cardiovascular disease; HOMA-IR, homeostasis model of assessment—insulin resistance; HTN, hypertension; LDL-C, low-density lipoprotein cholesterol; MetS, metabolic syndrome; TC, total cholesterol; TG, triglycerides; T2DM, type 2 diabetes mellitus.

**Table 4:** Associations between various baseline characteristics and the likelihood of sustained adherence to the Mediterranean diet over 10 years of follow-up.

Total Sample (*n* = 2583)	OR	95% CI	*p*-Value
Age (per 1 year increase)	0.942	0.927–0.956	<0.001
Sex (males vs. females)	0.163	0.110–0.241	<0.001
SES (medium vs. low) (high vs. low)	1.121 1.404	0.601–2.093 0.744–2.649	0.719 0.295
Smoking (current smokers vs. non-smokers)	0.914	0.657–1.272	0.595
BMI (per 1 kg/m^2^ increase)	0.756	0.715–0.799	<0.001
Physical activity (per 100 MET-min/week increase)	1.000	0.999–1.020	0.067
**<40 years (*n* = 1304)**	**OR**	**95% CI**	***p*-Value**
Sex (males vs. females)	0.192	0.128–0.288	<0.001
SES (medium vs. low) (high vs. low)	0.686 0.729	0.304–1.550 0.320–1.663	0.365 0.453
Smoking (current smokers vs. non-smokers)	0.954	0.669–1.361	0.797
BMI (per 1 kg/m^2^ increase)	0.777	0.734–0.822	<0.001
Physical activity (per 100 MET-min/week increase)	1.014	1.000–1.025	0.012
**≥40 years (*n* = 1279)**	**OR**	**95% CI**	***p*-Value**
Sex (males vs. females)	0.853	0.547–1.392	0.991
SES (medium vs. low) (high vs. low)	5.070 5.389	1.491–17.24 1.379–21.05	0.025 0.009
Smoking (current smokers vs. non-smokers)	0.674	0.289–1.576	0.363
BMI (per 1 kg/m^2^ increase)	0.521	0.430–0.631	<0.001
Physical activity (per 100 MET-min/week increase)	1.000	0.999–1.039	0.650

Results are presented as odds ratios with their 95% confidence intervals and *p*-values, as derived from multiple logistic regression analysis models. The high–high Mediterranean diet trajectory (high adherence to the Mediterranean diet, i.e., equal or above the median MedDietScore value, both at baseline and the 10-year follow-up evaluation) served as the dependent variable. BMI, body mass index; CI, confidence interval; MET-min/week, weekly minutes of metabolic equivalents of tasks; OR, odds ratio; SES, socioeconomic status.

## Data Availability

The data that support the findings of this study are available from the ATTICA study but restrictions apply to the availability of these data, which were used under license for the current study, and so are not yet publicly available. Data are, however, available from the authors upon reasonable request and with permission of the ATTICA project.
